# Clinical attributes, treatment, and control in hypertension (CATCH)—a French and Italian longitudinal patient database study

**DOI:** 10.1186/s40885-015-0029-2

**Published:** 2015-08-10

**Authors:** David Wu, George Mansoor, Christian Kempf

**Affiliations:** Merck &Co., Inc., Kenilworth, NJ USA; Cegedim Strategic Data, Paris, France; Global Health Outcomes, Merck & Co., Inc., 600 Corporate Drive, CRB-205, Cokesbury, NJ 08833 USA

**Keywords:** Hypertension, Cardiovascular disease, Risk factor, Blood pressure, Treatment

## Abstract

**Introduction:**

Hypertension, a risk factor for cardiovascular disease (CVD), is frequently associated with other CVD risk factors. Despite recent improvement in blood pressure (BP) control in Europe, a substantial proportion of patients fail to achieve BP targets.

**Methods:**

This retrospective cohort study used longitudinal patient databases (LPDs) in France and Italy to examine CVD risk profiles, treatment patterns, and BP goal attainment in hypertensive patients treated in real-world clinical practice between 2007 and 2008. Overall, 147,964 and 140,189 eligible patients from LPDs in France and Italy, respectively, were prescribed an antihypertensive medication in 2007.

**Results:**

Among patients with hypertension with other risk factors (France 88 %, Italy 83 %), the most prevalent risk factors were being elderly (France 66.9 %, Italy 70. 9 %), followed by hypertension combined with dyslipidemia (France 36.7 %, Italy 23.9 %) and isolated systolic hypertension (France 32.5 %, Italy 24.2 %). The odds ratios for target BP attainment were significantly (*p* < 0.001) higher in patients with hypertension without other risk factors vs patients with hypertension with other risk factors (1.41 [95 % confidence interval 1.35, 1.48] in France; 1.38 [1.31, 1.46] in Italy). The odds of BP control were significantly lower for patients with vs patients without an associated CVD risk factor (range 0.54 to 1.10 France; 0.59 to 1.17 Italy).

**Discussion:**

This study demonstrates that the majority of treated hypertensive patients in France and Italy have at least one additional CVD risk factor. Despite treatment with antihypertensive medications, blood pressure attainment was substantially less optimal in patients with an associated CVD risk factor compared to patients without an associated CVD risk factor.

**Electronic supplementary material:**

The online version of this article (doi:10.1186/s40885-015-0029-2) contains supplementary material, which is available to authorized users.

## Introduction

The majority of hypertensive patients have concomitant cardiovascular disease (CVD) risk factors [1, 2], and CVD risk is higher among these hypertensive patients compared with the general population. Response to antihypertensive therapy may differ between those at high and low risk [3, 4]. For example, achieving blood pressure target may be more difficult for patients with concomitant cardiovascular risk factors or advanced age while needing more frequent therapy changes or dose adjustments [5]. Consequently, hypertension treatment guidelines have been oriented towards individual management, taking into account all concomitant risk factors rather than focusing only on blood pressure measures for determining the need and type of treatment [6]. Both the 2007 and 2013 European guidelines have indicated that diagnosis and management of hypertension should be informed by the quantification of total CVD risk and interpreted using a physician’s knowledge and experience [7, 8]. In addition to lifestyle changes, the 2013 evidence-based guidelines recommend therapeutic approaches for all patients, including the elderly and patients with diabetes, cardiovascular disease, cerebrovascular disease, or renal disease to achieve a blood pressure target of <140/90 mmHg [8]. The 2007 evidence-based guidelines recommend a lower blood pressure target of 130/80 mmHg for patients with diabetes; patients with cerebrovascular, heart, or renal disease; obese patients, and those with multiple risk factors [7].

Despite overall improvement in blood pressure treatment and control over the past decades in Europe and the USA [9, 10], a substantial proportion of patients are still not achieving their blood pressure goals [3, 11, 12]. A cross-sectional study conducted in 12 European countries showed that only 38.8 % of hypertensive patients achieved the blood pressure target of 140/90 mmHg in real-life clinical practice [11]. Belletti et al. showed that compared to patients with hypertension and no additional cardiovascular risk factors, adjusted odds ratios for attainment of target blood pressure were significantly lower in patients with associated risk factors [5]. Wong et al. showed that although treatment rates were lowest in lower risk individuals and highest in patients with cardiovascular disease, blood pressure (BP) control rates were best in lower risk patients and worst in the higher risk patients [4]. This indicates that the level of unmet medical need and suboptimal care may differ across patient populations.

To help better understand the remaining unmet needs in blood pressure treatment, we examined CVD risk profiles, treatment patterns, and blood pressure goal attainment in hypertensive patients treated in actual clinical practices in France and Italy. The specific study objectives were (1) to identify demographic characteristics and CVD risk profiles of adult hypertensive patients, (2) to examine and document antihypertension treatment patterns in segments of patient populations with and without hypertension with other risk factors, and (3) to assess blood pressure goal attainment rates in these patient segments.

## Methods

### Study design

This was a retrospective, observational, longitudinal, single-cohort database study conducted in primary health care practices in France and Italy. The study period was 1 January 2005 to 31 December 2008.

### Data source

Primary care data were extracted from Cegedim Strategic Data’s (CSD’s) proprietary, longitudinal patient databases (LPDs) in Italy and France. CSD’s LPDs hold anonymized electronic patient records collected from 700 participating general practices (GPs) in Italy who use the Millewin patient management software. CSD’s LPD in France holds similar data from 1200 participating GPs who use the Doc’Ware patient management software. The panels of volunteering participants are selected to be nationally representative according to geographical area, age, and gender. CSD’s LPDs contain patient-level prescriptions, diagnoses, demographics, and other medical data entered at the time of consultation by participating GPs and include routinely collected electronic health records for more than 800,000 patients in Italy and 1,600,000 patients in France. Information on diagnoses is encoded at the time of information collection, the codes being mapped on the tenth revision of the International Classification of Disease (ICD-10). Prescription data contain the dispensed drug name, the Anatomical Therapeutic Chemical (ATC) classification category, dose regimen, and prescription duration.

### Study population

The study population was defined as those aged 18 years or older at index date who fulfilled the following criteria during the index period (1 January 2007 to 31 December 2007, see Additional file 1: Figure S1): having a diagnosis of primary hypertension (ICD-10: I10) and at least one prescription for an antihypertensive medication (ATC codes C02, C03, C07, C08, and C09). In addition, patients had to have a minimum of 2 years of recorded activity in the database prior to the index date (baseline period, see Additional file 1: Figure S1) and at least 12 months of recorded activity in the database post index date and prior to study end on 31 December 2008 (follow-up period, see Additional file 1: Figure S1) to ensure that patients were longitudinally followed, including sufficient history and follow-up to meet our study objective. The index date, which was the date of inclusion for each patient, was determined to be the date of first observed prescription for any antihypertensive drug during the index period (Additional file 1: Figure S1). Patients were excluded if they had a diagnosis of secondary hypertension (ICD-10: I15) in the 2 years prior to the index date.

### Data collection and organization

Patient-level demographic characteristics and clinical data extracted from the databases at index date as baseline measures were as follows: demographics and vital signs (blood pressure and last laboratory result prior to the index date for lipids, glycosylated hemoglobin (HbA1c), and serum creatinine). Index medication was defined as the last prescription of antihypertensive medication(s) prior to or on the index date. Medical history data were extracted during the baseline period according to ICD-10 classification. These data were used to stratify the study population into non-mutually exclusive patient groups based on specific CVD risk profiles (definitions given in Additional file 2: Table S1) [7].

The last measure of blood pressure prior to the end of the follow-up period was collected. Treatment data (including antihypertensive medication name, daily dose, and duration) were also collected during the follow-up period and were stratified into monotherapies, fixed-dose combination medications, and free-dose combination therapies. The following medications were defined as categories of monotherapy: centrally acting agents, diuretics, beta blockers, calcium-channel blockers (CCBs), angiotensin-converting enzyme inhibitors (ACEIs), or angiotensin II receptor blockers (ARBs). Fixed-dose combination medications were defined as diuretic + beta blocker, beta blocker + CCB, ACEI + diuretic, ACEI + CCB, ARB + diuretic, or ARB + CCB. Free-dose combinations were those combination therapies that were not fixed-dose.

The study population was stratified into two treatment groups: treatment-naive and previously treated. The treatment-naive patient group had no antihypertensive medication prescribed during the baseline period, and the index prescription was regarded as the first-line therapy. Those in the previously treated group were prescribed at least one antihypertensive medication during the baseline period.

### Data analysis

Descriptive statistics were employed using SAS 9.2 software to assess patient characteristics, clinical profile, treatment pattern, and blood pressure control. No sample size calculation was necessary; data collection was exhaustive given the defined criteria. Categorical data were summarized by sample size and/or percentage (compared to the size of completed data). Mean, median, standard deviation, and range were reported for continuous variables. Univariate binary logistic regressions were performed to predict the achievement of blood pressure goal with selected predictors or covariates in the survey. Estimated odds ratios (OR) were presented with 95 % confidence intervals in order to assess the relationship between the achievement of blood pressure goal and the presence of the factor, one by one. Next, 11 predictor variables were selected for France and 8 predictor variables for Italy in the multivariate model when *p* values were less than 0.05. A stepwise multivariate logistic regression was performed to keep only the variables significant at the 0.05 level, and adjusted ORs with 95 % CI were then calculated.

The last blood pressure measurement within the follow-up period was used to identify the blood pressure control rates based on levels defined in the 2007 ESH/ESC treatment guidelines in each defined patient segment [7]. The 2007 guidelines were utilized since the index dates for the study population were 1 January 2007 to 31 December 2007. The index antihypertensive medication prescription for each patient was compared with subsequent prescription(s) to assess treatment patterns—switches, add-ons, and discontinuation—in both treatment groups. Switch was defined as the replacement of the index medication with another antihypertensive medication; only the first observed switch was considered to capture the initial switch decision. Add-on was defined as the addition of any antihypertensive medication to the index medication during the follow-up period (a repeat prescription for the index medication in addition to prescription of another drug on a unique script). Discontinuation was defined as terminating therapy with the index medication and not receiving another prescription for a period of at least twice the expected duration of the previous prescription (assumed days of medication supplied).

## Results

### Prevalence of HTN with other risk factors by subpopulation and baseline characteristic

A total of 147,964 and 140,189 patients from LPDs in France and Italy, respectively, were prescribed an antihypertensive medication during the index period and were eligible for the study. Baseline patient demographics and clinical characteristics are reported in Additional file 3: Table S2. Gender, age, BMI, SBP, and DBP distributions were similar among patients in both countries. The mean age of patients was 66 years in France and 68 years in Italy, with slightly more patients being women in both countries (France 52.2 %, Italy 56.6 %). The majority of patients were defined as having hypertension with other risk factors (France 88 %, Italy 83 %). Among them, elderly patients with hypertension were the largest prevalent patient segment in each country (France 66.9 %, Italy 70.9 %), followed by patients with hypertension and dyslipidemia (France 36.7 %, Italy 23.9 %) and patients with isolated systolic hypertension (France 32.5 %, Italy 24.2 %).

### Antihypertensive treatment patterns

#### Index medication patterns in naive and treatment-experienced patients

In France, angiotensin II receptor blockers (ARBs, 32.8 %) were the medication group most commonly prescribed at index date among treatment-naive patients (see Table 1). Calcium-channel blockers (CCBs, 12.9 %), beta blockers (12.7 %), angiotensin-converting enzyme inhibitors (ACEIs, 10.3 %), and diuretics (9.4 %) were the next most commonly prescribed groups of medication among these patients. Among previously treated patients, treatment combinations not classified in the fixed-dose combinations were the most prescribed group (41.6 %), followed by ARBs alone (13.0 %), fixed-dose combinations of ARB + diuretic (10.5 %), beta blockers alone (8.9 %), and calcium-channel blockers alone (6.0 %).

**Table 1:** Prescription pattern at index date

Medication category	France			Italy		
	Naive patients (*n* = 9563)	Previously treated patients (*n* = 138,401)	Total (*n* = 147,964)	Naive patients (*n* = 9027)	Previously treated patients (*n* = 131,162)	Total (*n* = 140,189)
ACEI	988 (10.3 %)	7651 (5.5 %)	8639 (5.8 %)	3438 (38.1 %)	25,593 (19.5 %)	29,031 (20.7 %)
ARB	3132 (32.8 %)	17,937 (13.0 %)	21,069 (14.2 %)	1242 (13.8 %)	13,663 (10.4 %)	14,905 (10.6 %)
Beta blockers	1210 (12.7 %)	12,308 (8.9 %)	13,518 (9.1 %)	1283 (14.2 %)	13,444 (10.2 %)	14,727 (10.5 %)
Centrally acting agents	312 (3.3 %)	1673 (1.2 %)	1985 (1.3 %)	40 (0.4 %)	633 (0.5 %)	673 (0.5 %)
CCB	1238 (12.9 %)	8241 (6.0 %)	9479 (6.4 %)	901 (10.0 %)	15,681 (12.0 %)	16,582 (11.8 %)
Diuretics	902 (9.4 %)	7131 (5.2 %)	8033 (5.4 %)	89 (1.0 %)	312 (0.2 %)	401 (0.3 %)
ACEI + diuretic^a^	291 (3.0 %)	5614 (4.1 %)	5905 (4.0 %)	1026 (11.4 %)	18,420 (14.0 %)	19,446 (13.9 %)
ARB + CCB^a^	17 (0.2 %)	13 (0.0 %)	30 (0.0 %)	–	–	–
ARB + diuretic^a^	428 (4.5 %)	14,487 (10.5 %)	14,915 (10.1 %)	539 (6.0 %)	14,079 (10.7 %)	14,618 (10.4 %)
Beta blocker + CCB^a^	10 (0.1 %)	1037 (0.7 %)	1047 (0.7 %)	0 (0.0 %)	3 (0.0 %)	3 (0.0 %)
Beta blocker + diuretic^a^	231 (2.4 %)	3920 (2.8 %)	4151 (2.8 %)	39 (0.4 %)	96 (0.1 %)	135 (0.1 %)
CCB + ACEI^a^	45 (0.5 %)	766 (0.6 %)	811 (0.5 %)	–	–	–
Free-dose combinations^a^	759 (7.9 %)	57,623 (41.6 %)	58,382 (39.5 %)	430 (4.8 %)	29,238 (22.3 %)	29,668 (21.2 %)

*ACEI* angiotensin-converting enzyme inhibitors, *ARB* angiotensin II receptor blockers, *CCB* calcium-channel blockers

^a^Fixed-dose combinations

In Italy, ACEIs (38.1 %) were the most commonly prescribed index medication group among treatment-naive patients, followed by beta blockers alone (14.2 %), ARBs alone (13.8 %), fixed-dose combinations of ACEI + diuretic (11.4 %), calcium-channel blockers alone (10.0 %), and fixed-dose combinations of ARB + diuretic (6.0 %). Among previously treated patients, treatment combinations not classified in the fixed-dose combinations were the most prescribed group at index date (22.3 %), followed by ACEIs alone (19.5 %), fixed-dose combinations of ACEI + diuretic (14.0 %), calcium-channel blockers alone (12.0 %), fixed-dose combinations of ARB + diuretic (10.7 %), ARBs (10.4 %), and beta blockers alone (10.2 %).

#### Medication changes during follow-up period in naive and treatment-experienced patients

**Fig. 1:**
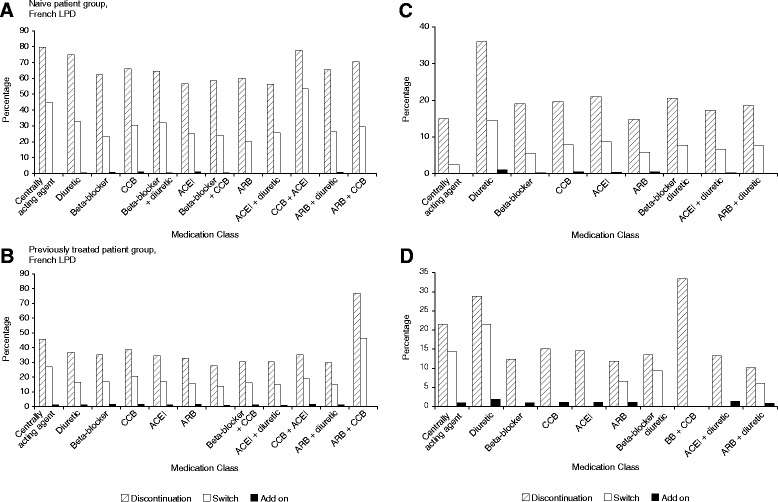
Changes in treatment for naive and for previously treated patients by treatment subgroup. **a** Treatment patterns in naive patients included in the French longitudinal patient database. **b** Treatment patterns in previously treated patients included in the French longitudinal patient database. **c** Treatment patterns in naive patients included in the Italian longitudinal patient database. **d** Treatment patterns in previously treated patients included in the French longitudinal patient database

With the exception of patients treated with fixed-dose combinations of ARB + CCB, treatment discontinuations were more frequent among treatment-naive French patients than among previously treated patients in all treatment groups. The difference ranged from 26.1 % for treated with fixed combinations of ACEI + diuretic to 42.8 % for patients treated with fixed-dose combinations of CCB + ACE at index date (Fig. 1a, b). Depending on the type of treatment prescribed at index date, treatment switches occurred between 20.0 % (beta blocker + CCB) and 53.3 % (CCB + ACEI) of treatment-naive patients and between 14.8 % (ARB + diuretic) and 46.2 % (ARB + CCB) of previously treated patients. Add-ons of a new class were prescribed in no more than 1.1 % of naive patients for all treatment groups and no more than 1.7 % of previously treated patients.

Among Italian patients, drug discontinuations ranged from 14.8 % for the ARB alone subgroup to 36 % for the diuretics alone subgroup in treatment-naive patients. Discontinuation rate varied from 10.2 to 33.3 % across index medication subgroups in previously treated patients. Treatment switches were generally less frequent than discontinuation in both treatment-naive patients (from 2.5 to 14.6 % across index medication subgroups) and previously treated patients (from 0 to 21.5 %).

### Target blood pressure attainment after 12 months follow-up

#### Target blood pressure attainment rate across subpopulations

**Fig. 2:**
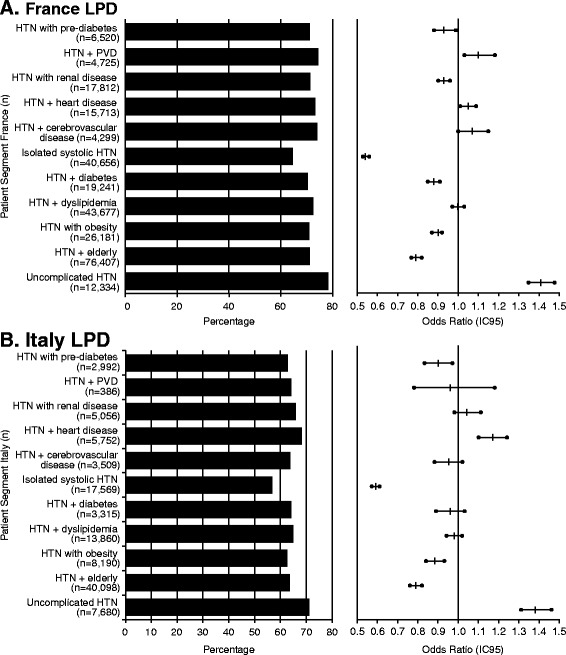
Blood pressure goal attainment across patient segments after 12 months of follow-up (missing data not included in analyses). **a** Goal attainment in patients included in the French longitudinal patient database. Forest plot was derived from univariate regression analysis and shows odds ratios and 95 % confidence intervals for attainment of BP target (140/90 mm Hg). **b** Goal attainment in patients included in the Italian longitudinal database. Forest plot was derived from univariate regression analysis and shows odds ratios and 95 % confidence intervals for attainment of BP target (140/90 mm Hg)

Attainment of target blood pressure after 12 months was determined for patients with a blood pressure value available in the LPDs during the follow-up period. The proportion of patients with these data available was 76.7 % for French patients and 39.1 % for Italian patients. The proportion of patients attaining target blood pressure after 12 months of follow-up was greatest in the segment of patients with hypertension without other risk factors and reached 78.4 % and 71.2 % of French and Italian patients, respectively (Fig. 2). In patients with hypertension with other risk factors, the overall proportion of target blood pressure attainment was 72.6 % in France and 65.1 % in Italy. These proportions ranged from 64.7 % to 74.5 % for French and from 56.8 % to 68.3 % for Italian patients across subpopulations.

#### Comparison of BP control across subpopulations

Based on the univariate logistic regression analysis, the odds ratios for target BP attainment were significantly (*p* < 0.001) higher in patients with hypertension without other risk factors vs patients with hypertension with other risk factors (1.41 [95 % confidence interval (CI) 1.35, 1.48] in France; 1.38 [1.31, 1.46] in Italy). The odds of BP control were significantly lower for patients with vs patients without an associated CVD risk factor (range 0.54 to 1.10 in France and 0.59 to 1.17 in Italy).

In France, multiple stepwise logistic regression (Table 2) showed that factors associated with lower odds of attaining blood pressure goal included being male >65 years (OR = 0.87; 95 % CI 0.84, 0.90), being female >65 years (OR = 0.82; 95 % CI 0.79, 0.85), isolated hypertension (OR = 0.76; 95 % CI 0.74, 0.79), having obesity (OR = 0.92; 95 % CI 0.89, 0.95) or diabetes (OR = 0.93; 95 % CI 0.90, 0.97) comorbid with hypertension, and higher baseline diastolic (OR = 0.99; 95 % CI 0.99, 0.99) and systolic blood pressure (OR = 0.96; 95 % CI 0.96, 0.96). Conversely, patients having CVD comorbid with hypertension (OR = 1.09; 95 % CI 1.01, 1.17), heart disease comorbid with hypertension (OR = 1.06; 95 % CI 1.02, 1.11), and peripheral vascular disease comorbid with hypertension (OR = 1.13; 95 % CI 1.05, 1.21) and drug-naive patients (OR = 1.76; 95 % CI 1.65, 1.87) had higher odds of reaching blood pressure goals in France. In Italy, factors significantly associated with lower odds of attaining blood pressure goal included being female >65 years (OR = 0.90; 95 % CI 0.86, 0.94), hypertension combined with obesity (OR = 0.86; 95 % CI 0.81, 0.90), isolated systolic hypertension (OR = 0.80; 95 % CI 0.76, 0.84), and higher baseline diastolic (OR = 0.99; 95 % CI 0.99, 1.00) and systolic blood pressure (OR = 0.96; 95 % CI 0.96, 0.97), whereas having comorbid heart disease with hypertension (OR = 1.17; 95 % CI 1.09, 1.25), having hypertension without other risk factors (OR = 1.08; 95 % CI 1.01, 1.17), or being drug naive (OR = 1.91; 95 % CI 1.75, 2.08) increased the odds of achieving blood pressure target (Table 2).

**Table 2:** Factors associated with blood pressure goal attainment based on multivariate stepwise logistic regression model (*N* = 107,353)

	*n*	OR	95 % CI	*p* value
France				
Female >65 years	31,777	0.82	0.79, 0.85	<0.001
Male >55 years	40,627	0.87	0.84, 0.90	<0.001
HTN + obesity	25,141	0.92	0.89, 0.95	<0.001
HTN + diabetes	18,316	0.93	0.90, 0.97	<0.001
Isolated systolic HTN	40,656	0.76	0.74, 0.79	<0.001
HTN + CVD	4104	1.09	1.01, 1.17	<0.033
HTN + heart disease	14,944	1.06	1.02, 1.11	<0.006
HRN + PVD	4534	1.13	1.05, 1.21	<0.001
Drug naive	6251	1.76	1.65, 1.87	<0.001
BL diastolic BP	107,353	0.99	0.99, 0.99	<0.001
BL systolic BP	107,353	0.96	0.96, 0.96	<0.001
Italy				
Female >65 years	18,080	0.9	0.86, 0.94	<0.001
HTN + obesity	7365	0.86	0.81, 0.90	<0.001
Isolated systolic HTN	15,977	0.8	0.76, 0.84	<0.001
HTN + heart disease	4813	1.17	1.09, 1.25	<0.001
Uncomplicated HTN	5682	1.08	1.01, 1.17	<0.033
Drug naive	3134	1.91	1.75, 2.08	<0.001
BL diastolic BP	45,060	0.99	0.99, 1.00	<0.001
BL systolic BP	45,060	0.96	0.96, 0.97	<0.001

*BL* baseline, *BP* blood pressure, *CI* confidence interval, *CVD* cardiovascular disease, *HTN* hypertension, *OR* odds ratio, *PVD* peripheral vascular disease

## Discussion

The results of this analysis showed that, consistent with previous findings in the general population, more than four out five patients in France and Italy with hypertension who are already treated by general practitioners had at least one other cardiovascular disease risk factor. Yet, in this population, attainment of target blood pressure after 1 year of treatment was better in those with hypertension without other risk factors than in patients with hypertension with other risk factors [3, 5, 13]. In addition, this analysis identified that most treatment-naive patients in France and Italy were prescribed a single class of drug, and previously treated patients in both countries were being managed with combinations of drugs not considered fixed-dose combinations.

As noted in the 2013 guidelines and in other reports from industrialized countries, only a small proportion of patients with hypertension suffers from elevated BP alone [8]. In the current analysis, older age was by far the most prevalent associated risk factor, followed by dyslipidemia and isolated systolic hypertension. Our findings are consistent with other reports in which the prevalence of patients with hypertension associated with at least one risk factor ranged between ~74 % and 95 % in industrialized countries. In those studies, additional risk factors of patients with hypertension were similar to those in the present analysis including age ≥65 years and a high prevalence of obesity and dyslipidemia, especially in women [3, 5, 13]. The addition of one or more cardiovascular risk factors to BP may lead to a total CV risk profile that is greater than the sum of its individual elements. That said, among treated hypertensive patients, there are certain subgroups of hypertensive patients with other risk factors that are less optimally controlled.

In this study, the results showed that attainment of target blood pressure after 1 year of treatment was better in those with hypertension without other risk factors than in the patient segments with hypertension with other risk factors. These results substantiate those of the PRATIK study and the Wong et al. study, which showed that patients at high CVD risk were less likely to be controlled for hypertension than patients at lower CVD risk [4, 14]. In the current analysis, despite the many different treatment options available, the majority of patients in hypertension segments with additional risk factors did not achieve target blood pressure. These findings are also consistent with previous results showing poor attainment of target blood pressure using similar assessment criteria. Estimates suggest that 53.5 % of those with hypertension in the USA had inadequately controlled blood pressure [15]. Studies in Italy and Europe indicate that more than 60 % of hypertension patients treated with antihypertensive medications failed to reach blood pressure control [11, 12]. In the PRATIK and Wong studies, higher risk individuals were more likely to be treated with monotherapy than with combination therapies, which may account for the lower likelihood of attainment of BP target [4, 14].

Current guidelines state that diuretics, beta blockers, calcium antagonists, ACEIs, and ARBs are all appropriate medications for the initiation and maintenance of antihypertensive treatment, either as monotherapy or in some combinations [8]. More than one third of all treatment-naive patients were prescribed ARBs (France 32.8 %) and ACEIs (Italy 38.4 %). This may be related to a large proportion of patients with associated metabolic disorders and the possible contraindication of diuretics in patients with metabolic syndrome [16]. Physician prescription patterns differed for treatment-naive patients and previously treated patients in the two countries. Among treatment-naive patients in France, most patients were prescribed a single class of drug, mainly ARBs, followed by CCBs, beta blockers, ACEIs, and diuretics. Previously treated patients were mostly prescribed free combinations, a choice that might be a consequence of a previously inadequate patient response when using monotherapy or fixed-dose combinations on difficult-to-manage subpopulations of patients. In addition, the current findings can be paralleled with the lower proportion of previously treated patients who were prescribed treatment combinations in Italy. A relatively low proportion of index medication changes and discontinuations were observed among the previously treated patients compared with the treatment-naive patients in France. This might be a consequence of historic (prior to index date) therapeutic changes which made the previously treated subpopulation generally more stable with regard to their treatments during the follow-up period. Treatment-naive patients in Italy generally had more index medication discontinuations but less treatment changes than previously treated patients. Given that the majority of study patients with hypertension with other risk factors do not reach their blood pressure goal and that there is relatively little switching, it would appear that physicians were not fully utilizing recommended blood pressure targets to inform decisions about necessary changes in antihypertensive medication. Further assessment of this patient population with regard to identifying factors independently associated with achieving blood pressure goal might be an appropriate next step and could be used to better inform physicians in general clinical practice.

Factors such as age, compliance, number of concomitant medications, and disease severity, among others, all play a role in creating individual disease states. The observation that patients with cardiovascular conditions comorbid with hypertension had higher odds of attaining blood pressure target was somewhat unexpected. One can speculate that these patients had modified their lifestyle based on their current disease state and/or were being treated for their comorbid conditions, resulting in more frequent doctor visits, more aggressive treatment, and potentially better follow-up. Factors associated with lower odds of attaining blood pressure goals included increased age in males and females, and isolated systolic hypertension. Since isolated hypertension accounts for 87 % of cases in older adults, these results were not surprising [17]. Patients with other conditions, such as older age, diabetes, and obesity, may not be perceived as having a need for aggressive treatment, leading to clinical inertia. More aggressive and/or more targeted treatment options may be needed for these patients in order for them to achieve their target blood pressure.

Importantly, there may be multiple challenges present in treating patients with certain comorbidities along with hypertension. For example, despite the availability of several antihypertensive medications (including fixed-dose combinations intended to simplify dosing, reduce costs, and improve compliance [18]), prescribing multiple medications to treat comorbid conditions may lead to drug-drug interactions or compliance issues [19]; increasing age may result in a changing metabolic profile that impacts some drugs [20]; the increasing cost of medications may be overwhelming for some patients, causing them to stop taking medications; and/or fixed-dose combinations may not provide the appropriate dose of one of the component medications to meet an individual’s needs. The potential challenges of using combination therapies for the management of some hypertensive patients with additional risk factors might indicate the need for future innovative drugs specialized for such subpopulations. Bearing this in mind, one must treat the individual, not the individual symptoms.

Due to the period of study, the renin inhibitor drug class was not considered in this study and, consequently, no assessment can be made with regard to this drug class. Furthermore, these study results are subject to the quality and availability of the EMR data collected by the participating physicians. Only one BP measurement closest to the end of the follow-up period was used, which may have created some challenges in determining BP control. However, most patients would have been treated for an extended period of time at the end of follow-up, and BP control should have been established. An additional limitation of this study was that a relatively imbalanced proportion of patients were lost to follow-up in the two populations (about 25 % in the French and 60 % in the Italian), and it is difficult to tell if the data were randomly or systematically lost. However, this is a reflection of the real-world environment in which the study was conducted and indicates a greater need for adherence to guidelines, better quality of control and recording of patient data regardless of outcome, and greater follow-up with patients. Given the limitations of the clinical settings where these data were collected, generalization of these findings to the wider population should be made with caution.

## Conclusion

This study confirms that the majority of treated hypertensive patients in France and Italy have at least one associated additional CVD risk factor. Despite many treatment options available, attainment of blood pressure targets was substantially less optimal in patients with at least one associated CVD risk factor compared with patients without any associated CVD risk factors. The findings from this study can be used by health care providers to help identify gaps in quality of care for patients being treated for hypertension and to review appropriate interventions and follow-up patterns.

## Additional files

Additional file 1: Figure S1. Study schematic. (ZIP 297 kb) Additional file 2: Table S1. Data collected in longitudinal patient databases and definition of patient segments. (DOC 47 kb) Additional file 3: Table S2. Baseline Patient Demographics and Clinical Profile*. (DOC 60 kb)

## Abbreviations

ACEIs: angiotensin-converting enzyme inhibitors
ARBs: angiotensin II receptor blockers
ATC: Anatomical Therapeutic Chemical
BP: blood pressure
CCBs: calcium-channel blockers
CI: confidence interval
CSD’s: Cegedim Strategic Data’s
CVD: cardiovascular disease
HbA1c: glycosylated hemoglobin
ICD-10: tenth revision of the International Classification of Disease
LPDs: longitudinal patient databases
OR: odds ratios
