# Supplementary material for: Clinical attributes, treatment, and control in hypertension (CATCH)—a French and Italian longitudinal patient database study
Source: Clin Hypertens. 2015 Aug 10;21:18. doi: 10.1186/s40885-015-0029-2 (PMC4750805; doi:10.1186/s40885-015-0029-2)
Supplement: Additional file 2: Table S1. — Data collected in longitudinal patient databases and definition of patient segments. (DOC 47 kb) [file 40885_2015_29_MOESM2_ESM.doc]

Supplemental Table 1. Data collected in longitudinal patient databases and definition of patient segments

| **Patient segment** | **Documented conditions during baseline period (ICD10 codes)** |
| --- | --- |
| Uncomplicated hypertension (HTN) | None |
| Complicated hypertension (HTN) subclasses | |
| Elderly | Age: man >55, women >65 |
| Diabetes | Diagnosis of diabetes (E10-E14, H35) |
| Cerebrovascular disease | Ischaemic stroke, cerebral haemorrhage, transient ischaemic attack; (I60-I69) |
| Heart disease | Myocardial infarction, angina, coronary revascularisation, heart failure (I20-I25, I50) |
| Renal disease | Diabetic nephropathy, renal impairment (N00-N39) |
| Peripheral vascular disease (PVD) | PVD (I73.9) |
| Isolated systolic hypertension | Systolic BP ≥140 mmHg and Diastolic BP <90 mmHg |
| Prediabetes | Fasting plasma glucose test in the range [110 mg/dl ; 126 mg/dl] |
| Obesity | BMI ≥30 kg/m² (E88.81) |
| Dyslipidemia | Diagnosis of dyslipidemia (E78) |
