# Supplementary material for: Clinical attributes, treatment, and control in hypertension (CATCH)—a French and Italian longitudinal patient database study
Source: Clin Hypertens. 2015 Aug 10;21:18. doi: 10.1186/s40885-015-0029-2 (PMC4750805; doi:10.1186/s40885-015-0029-2)
Supplement: Additional file 3: Table S2. — Baseline Patient Demographics and Clinical Profile*. (DOC 60 kb) [file 40885_2015_29_MOESM3_ESM.doc]

Supplemental Table 2: Baseline Patient Demographics and Clinical Profile*

| **Characteristic** | | **Overall** | Uncomplicated Hypertension (HTN) | HTN + elderly | HTN + dyslipi-daemia | Isolated systolic HTN | HTN + obesity | HTN + heart disease | HTN + diabetes | HTN + renal disease | HTN + cerebro-vascular disease | HTN + pre-diabetes | HTN + Peripheral vascular disease |
| --- | --- | --- | --- | --- | --- | --- | --- | --- | --- | --- | --- | --- | --- |
| France | Patient number (% of distribution) | **N=147,964** | 17,885 (12.1%) | 98,982 (66.9%) | 54,275 (36.7%) | 48,091 (32.5%) | 31,911 (21.6%) | 20,052 (13.6%) | 24,297 (16.4%) | 21,593 (14.6%) | 5,550 (3.8%) | 7,839 (5.3%) | 5,875 (4.0%) |
|  | Sex - male (%) | 70,783 (47.8%) | 5,570 (31.1%) | 55,493 (56.1%) | 27,077 (49.9%) | 22,750 (47.3%) | 15,405 (48.3%) | 11,234 (56.0%) | 13,737 (56.5%) | 7,989 (37.0%) | 3,048 (54.9%) | 4,539 (57.9%) | 2,052 (34.9%) |
|  | Age – mean ( SD) | 66 (12.5) | 51 (8.1) | 73 (8.7) | 68 (10.9) | 69 (11.6) | 63 (11.6) | 72 (11.2) | 67 (10.7) | 76 (8.9) | 73 (10.8) | 67 (11.0) | 70 (11.7) |
|  | Body mass index (kg/m²) – mean (SD) | 28.1 (5.11) | 25.1 (2.95) | 27.7 (4.65) | 28.2 (4.84) | 28.1 (5.10) | 34.1 (3.87) | 28.2 (5.01) | 30.3 (5.31) | 25.5 (4.07) | 27.3 (4.79) | 29.4 (5.02) | 28.4 (5.52) |
|  | Systolic blood pressure (mmHg) – mean (SD) | 140 (15.4) | 137 (17.6) | 140 (15.2) | 139 (14.8) | 146 (9.5) | 140 (15.1) | 139 (15.4) | 140 (15.6) | 140 (15.4) | 139 (15.3) | 140 (15.0) | 139 (14.9) |
|  | Dystolic Blood Pressure (mmHg) – mean (SD) | 80 (9.4) | 83 (11.9) | 79 (8.7) | 79 (8.8) | 78 (5.3) | 81 (9.5) | 78 (8.8) | 79 (9.0) | 78 (8.6) | 78 (8.8) | 80 (9.1) | 79 (8.6) |
|  | HbA1C (%) – mean (SD) | 6.8 (1.32) | 6.0 (0.96) | 6.8 (1.24) | 6.8 (1.25) | 6.9 (1.27) | 6.9 (1.33) | 6.9 (1.31) | 7.0 (1.33) | 6.8 (1.26) | 6.9 (1.30) | 6.3 (0.77) | 6.7 (1.25) |
| Italy | Patient number (% of distribution) | **N=140,189** | 24,097 (17.2%) | 99,383 (70.9%) | 33,460 (23.9%) | 33,974 (24.2%) | 15,757 (11.2%) | 14,640 (10.4%) | 7,026 (5.0%) | 9,257 (6.6%) | 8,219 (5.9%) | 6,109 (4.4%) | 858 (0.6%) |
|  | Sex - male (%) | 60,804 (43.4%) | 8,092 (33.6%) | 48,252 (48.6%) | 15,564 (46.5%) | 13,462 (39.6%) | 6,611 (42.0%) | 7,840 (53.6%) | 3,111 (44.3%) | 3,007 (32.5%) | 3,797 (46.2%) | 2,974 (48.7%) | 477 (55.6%) |
|  | Age – mean ( SD) | 68 (12.5) | 52 (7.9) | 74 (8.6) | 70 (9.8) | 72 (10.5) | 65 (11.5) | 74 (10.3) | 70 (10.7) | 77 (8.5) | 75 (9.6) | 70 (10.5) | 73 (9.6) |
|  | Body mass index (kg/m²) – mean (SD) | 28.9 (5.11) | 26.0 (2.63) | 28.4 (4.75) | 28.9 (4.72) | 28.7 (4.96) | 34.2 (3.97) | 28.6 (4.71) | 30.5 (5.62) | 26.4 (4.21) | 27.8 (4.45) | 29.8 (5.15) | 28.2 (4.57) |
|  | Systolic blood pressure (mmHg) – mean (SD) | 141 (16.7) | 138 (17.2) | 141 (16.8) | 140 (16.6) | 148 (10.5) | 141 (16.5) | 139 (17.1) | 141 (16.5) | 140 (17.0) | 139 (17.1) | 142 (16.6) | 141 (16.5) |
|  | Dystolic Blood Pressure (mmHg) – mean (SD) | 82 (9.4) | 87 (10.2) | 81 (8.9) | 81 (9.0) | 79 (5.5) | 84 (9.6) | 79 (8.8) | 81 (8.8) | 79 (8.6) | 80 (9.1) | 82 (9.1) | 80 (8.2) |
|  | HbA1C (%) – mean (SD) | 7.0 (1.44) | 6.9 (1.87) | 7.0 (1.37) | 7.2 (1.46) | 7.1 (1.40) | 7.1 (1.42) | 7.2 (1.42) | 7.3 (1.43) | 7.0 (1.36) | 7.2 (1.42) | 6.4 (0.92) | 7.0 (1.45) |

*^1^ A given patient can belong to several segments*

*Except otherwise stated results are presented in the form N (%)*
